# Prognostic Value and Therapeutic Potential of CBX Family Members in Ovarian Cancer

**DOI:** 10.3389/fcell.2022.832354

**Published:** 2022-01-27

**Authors:** Kuan Hu, Lei Yao, Zhijie Xu, Yuanliang Yan, Juanni Li

**Affiliations:** ^1^ Department of Hepatobiliary Surgery, Xiangya Hospital, Central South University, Changsha, China; ^2^ Department of Pathology, Xiangya Hospital, Central South University, Changsha, China; ^3^ Department of Pharmacy, Xiangya Hospital, Central South University, Changsha, China; ^4^ National Clinical Research Center for Geriatric Disorders, Xiangya Hospital, Central South University, Changsha, China

**Keywords:** CBX family, ovarian cancer, expression profiles, prognosis, chemoresistance

## Abstract

**Background:** Ovarian cancer (OV) is one of the common malignant tumors and has a poor prognosis. Chromobox (CBX) family proteins are critical components of epigenetic regulation complexes that repress target genes transcriptionally *via* chromatin modification. Some studies have investigated the function specifications among several CBXs members in multiple cancer types, however, little is known about the functions and prognostic roles of distinct CBXs family proteins in ovarian cancer.

**Methods:** In this study, several bioinformatics databases and *in vitro* experiments were used to analyze the expression profiles, prognostic values, and therapeutic potential of the CBXs family (CBX1-8) in ovarian cancer.

**Results:** It was found that higher expression of CBX3/8 and lower expression of CBX1/6/7 were detected in OV tissues. CBX2/4/5/8 were significantly correlated with individual cancer stages of OV. The expression of CBX1/2/3 were all significantly associated with worse overall survival (OS) and progression-free survival (PFS) for OV patients, whereas the expression of other five CBXs members showed either irrelevant (CBX5 and CBX8) or inconsistent (CBX4, CBX6, and CBX7) results for both OS and PFS in OV. These results showed that only CBX3 had consistent results in expression and prognosis. Further cell experiments also showed that CBX3 promoted the proliferation of ovarian cancer cells. CBX3 was highly expressed in chemoresistant OV tissues. These results indicated that CBX3 was the most likely prognostic indicator and new therapeutic target in OV. Furthermore, gene enrichment analysis suggests that the CBXs family was primarily involved in mast cell activation and mast cell mediated immunity. Individual CBXs members were associated with varying degrees of the infiltration of immune cells, especially B cells. Finally, a high genetic alteration rate of CBXs family (39%) was observed in OV. The low methylation status of CBX3/8 in OV may be associated with their high expression levels.

**Conclusions:** Taken together, these findings exhibited the pivotal value of CBXs family members (especially CBX3) in the prognosis and chemoresistance of ovarian cancer. Our results may provide new insight to explore new prognostic biomarkers and therapeutic targets for ovarian cancer.

## Introduction

Ovarian cancer is the most frequent cause of death in patients with gynecological malignancy, with five-year survival rates of less than 45% (Webb and Jordan, 2017; Liu et al., 2019; Siegel et al., 2021). Because of its asymptomatic development and the lack of reliable diagnostic markers, patients with ovarian cancer are frequently diagnosed at an advanced stage (Scarlett and Conejo-Garcia, 2012). The current standardized therapy strategy for patients with ovarian cancer is optimal cytoreductive surgery and platinum-based chemotherapy (Gupta et al., 2019; Moufarrij et al., 2019). Despite ovarian cancers usually responding well to platinum-based first-line chemotherapy, the majority of patients relapses frequently and develop chemoresistance with poor prognosis (Lim and Ledger, 2016). Although many years of researches there is still a lack of an early diagnosis method enabling early detection and suitable for screening. Hence, there is an urgent need to find new diagnostic biomarkers and new therapeutic targets to improve the diagnostic and treatment efficacy of ovarian cancer. Therefore, in this study, we are aimed to explore novel biomarkers with diagnostic, prognostic potential as well as on the finding for the new targeted therapy.

Emerging evidence showed that aberration of epigenetic regulation played a critical role in the regulation and progression of ovarian cancer (Chen et al., 2011; Natanzon et al., 2018). Polycomb group (PcG) complexes, as a kind of epigenetic regulatory complexes, have been detected to be dysregulated in various cancers and participate in the tumorigenesis and progression of these cancers (Casa and Gabellini, 2012; Chan and Morey, 2019). The chromobox (CBX) proteins are the crucial components of PcG that are involved in the regulation of several critical biologic processes such as self-renewal of cancer stem cells and cell differentiation (Klauke et al., 2013; Gil and O’Loghlen, 2014). To date, eight members of the CBXs proteins have been identified and were further subdivided into two groups (Siegel et al., 2021): heterochromatin protein 1 (HP1) group including CBX1, CBX3, and CBX5, it consists of an N-terminal chromodomain and a C-terminal chromodomain and is necessary for DNA repair, gene silencing, and telomere function (Kwon and Workman, 2008; Morey et al., 2012). Webb and Jordan (2017) Polycomb (Pc) group including CBX2, CBX4, CBX6, CBX7, and CBX8, contains only a conserved N-terminal chromodomain and could regulate transcription of target genes through interaction with PcG complex (Wotton and Merrill, 2007; Di Croce and Helin, 2013). Different CBXs members were correlated with chromatin different parts and regulated specific transcription of target genes (Zhen et al., 2016; Zeng et al., 2018).

Existing evidence have reported abnormal expressions and prognostic values of several CBXs members in some cancer types such as hepatocellular carcinoma (HCC) and osteosarcoma (Wang et al., 2013; Ma et al., 2019). However, the roles of distinct CBX family members in the development and progression of ovarian cancer still remained incompletely understood. Based on the rapid development of second-generation sequencing technology and the establishment of a large number of databases, a comprehensive study of the CBXs family in ovarian cancer is the benefit to discover new biomarkers with diagnostic and prognostic potential and therapeutic targets for this deadly disease. In the present study, we analyzed the expression of CBXs members and their relations with clinical parameters in OV. Cell experiments were conducted to explore the role of CBX3 in the proliferation of ovarian cancer cells. Then, the correlation between CBX3 and OV chemoresistance was explored. In addition, the predicted functions and pathways that were associated with CBXs family and their 246 frequently altered neighbor genes were also been investigated. Finally, we further analyzed the relationship between CBXs family and prognosis/immune infiltration/methylation in ovarian cancer. Our findings suggested that several CBXs family members might be considered potential biomarkers for prognosis and therapeutic targets in ovarian cancer.

## Materials and Methods

### ONCOMINE

The ONCOMINE database is a publicly accessible online cancer microarray database, it usually analyzes DNA or RNA sequences to promote discovery from the gene-wide expression analyses (Rhodes et al., 2004). In this work, we used this database to explore the differential mRNA expression of eight CBXs members between various cancer types and their corresponding normal tissues. The student’s t test was applied to compare the difference mRNA expression. Cut-off of *p*-value was defined as 0.05, the fold change was set up at 2 and the gene rank was defined as 10%. The databases used in this study were summarized in Supplementary Table S1.

### Gene Expression Profiling Interactive Analysis 2

The GEPIA2 (Gene Expression Profiling Interactive Analysis 2) database is a newly developed online tool that includes thousands of tumor samples and normal samples data from the TCGA (Tang et al., 2019). In the present study, this database was used to explore the expression levels of different CBXs members in ovarian cancer tissues and normal tissues.

### The Human Protein Atlas

The Human Protein Atlas is an online database that contains immunohistochemistry-based expression data for various cancer types. It aims to help researchers discover the expression patterns of specific proteins expressed in specific cancer types and thus identify clinically useful biomarkers (Asplund et al., 2012; Yan et al., 2019). In this analysis, we compared the protein expression levels of different CBXs members between ovarian serous cystadenocarcinoma tissues and normal ovarian stromal tissues by immunohistochemistry image.

### UALCAN

UALCAN is an interactive web resource based on TCGA datasets. it can analyze mRNA expression of specific genes between tumor tissues and normal tissues and the association of its expression with several clinicopathological characteristics such as tumor grade and individual cancer stages (Chandrashekar et al., 2017). In this work, we choose the “individual cancer stages” model to analyze the relations between the mRNA expression of different CBXs members and clinicopathological parameters in ovarian cancer. The student’s t test was applied and *p* < 0.05 was considered statistically significant.

### Kaplan–Meier Plotter

The Kaplan–Meier Plotter is a web tool that information about the association of specific gene expression with the survival of patients with ovarian cancer, breast cancer, lung cancer, gastric cancer, and liver cancer could be easily accessed (Deng et al., 2019; Li et al., 2020a). In this research, we used this database to investigate the association between the expression of CBXs members and overall survival (OS) and progression-free survival (PFS) for OV patients. A *p*-value less than 0.05 was considered statistically significant.

### cBioPortal

cBioPortal is a comprehensive online resource with visual and multidimensional cancer genomics and clinical data (Gao et al., 2013). In this work, the genetic alterations and mRNA expression of the CBXs family were analyzed. We obtained the mRNA expression z scores (RNA Seq V2 RSEM) using a z score threshold of ±0.7.

### WebGestalt

WebGestalt a comprehensive and flexible online resource that can conduct interactive web-based analysis (Liao et al., 2019). In this study, we applied this database to analyze Gene ontology (GO) enrichment and Kyoto Encyclopedia of Genes and Genomes (KEGG) pathway.

### Cytoscape

Cytoscape was applied to conduct functional integration on 246 frequently altered neighbor genes of the CBXs family screened from the cBioPortal database (Supplementary Table S2). The nodes’ size represented the degree values between these interacting proteins. The larger the circles, the higher the degree.

### TIMER2.0

The TIMER2.0 is an open service that can assess immune cell infiltration and their clinical significance for 32 cancer types (Li et al., 2020b). In this work, we used the “Gene module” tool to generate scatterplots to analyze the association between the expression of the CBXs members and the infiltration of six immune cells including B cells, CD8 + T cells, CD4 + T cells, macrophages, neutrophils and dendritic cells in ovarian cancer.

### DiseaseMeth2.0

DiseaseMeth2.0 is an accessible online resource that provides information on DNA methylation status in different kinds of human diseases, especially cancers (Lv et al., 2012; Xiong et al., 2017). In this analysis, we used this web to investigate the methylation status of the CBXs members between ovarian cancer tissues and normal tissues. *p* < 0.05 was considered as significant.

### Cell Lines

The human ovarian cancer cell lines SKOV3 and HO8910 were purchased from CRC/PUMC (Cell Resource Center, IBMS, CAMS/PUMC) and CCTCC (China Center for Type Culture Collection), respectively. These two cell lines were both cultured in 1640 RPMI medium supplemented with 10% fetal bovine serum.

### Transient Transfection

For transient transfections, CBX3 siRNAs or control siRNA (stB0002817A-1-5, Ribobio) were transfected into ovarian cancer cell lines using the Lipofectamine 3,000 reagent (Invitrogen) according to the manufacturer’s instruction. This siRNA of CBX3 product contained three different siRNA sequences.

### RNA Extraction and RT-qPCR

These processes were performed as previously described (Li et al., 2020c). The primer sequences of CBX3 used in this study were F: 5′ TGG​CCT​CCA​ACA​AAA​CTA​CA 3′; R: 5′ TCC​CAT​TCA​CTA​CAC​GTC​GA 3′. The GAPDH mRNA expression was applied as an internal reference for quantification.

### Western Blot

This process was performed as previously described (Li et al., 2020c). Briefly, the BCA protein assay kit (Thermo Fisher Scientific) was used to detect the concentration of protein. Then, 40 ug proteins were loaded and separated by SDS-PAGE, transferred onto polyvinylidene difluoride membranes, and blocked with 5% milk for about 1 hour. Next, membranes were incubated at 4°C overnight with the following primary antibodies: CBX3 (ab217999, Abcam) and GAPDH (sc-47724, Santa Cruz Biotechnology). The protein band intensities were evaluated by the Image Lab software (Bio-Rad, CA, USA). The GAPDH protein expression level was used as an internal control for normalization.

### CCK8 Assay

After indicated treatment, SKOV3 and HO8910 cells were seeded and cultured in 96-well plates at a density of 3,000 cells/well. The cell proliferation ability was evaluated using the Cell Counting Kit-8 (CCK-8, Dojindo, Kumamoto, Japan) according to the manufacturer’s instructions.

### Statistical Analyses

SPSS, version 18.0 (Chicago, United States) was used for the statistical analysis. The data were expressed as mean ± SD. The differences were determined by the student’s t test for two groups and the one-way analysis of variance (ANOVA) for multiple groups. Statistical significance was set at *p* < 0.05.

## Results

### Aberrant Expression of the CBXs Family in Ovarian Cancer Patients

**FIGURE 1:**
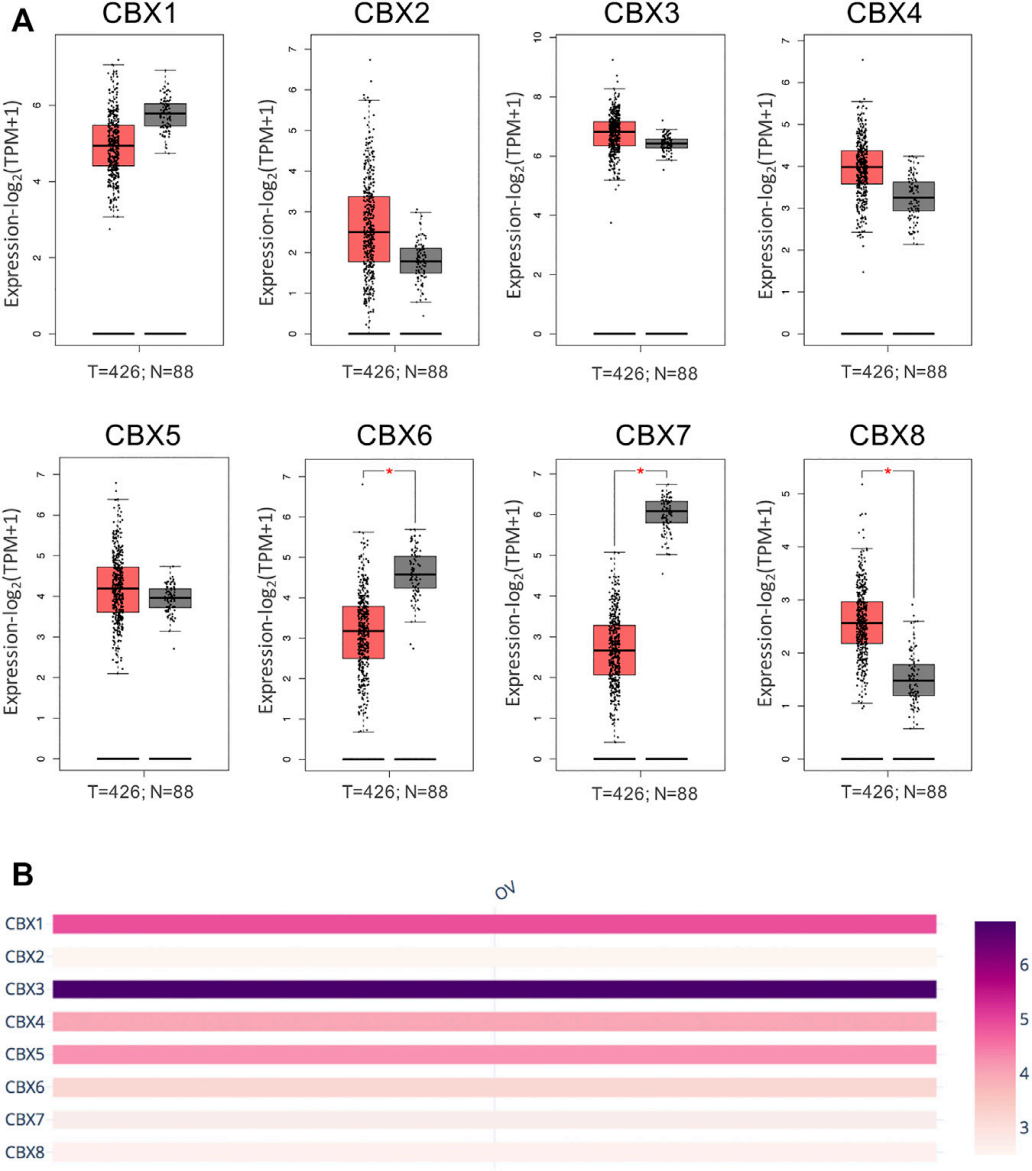
mRNA expression levels of the CBXs family members in OV tissues and normal ovarian tissues. **(A)** The mRNA expression profiles were obtained from the GEPIA2 databases. T represented ovarian cancer tissues; N represented normal ovarian tissues. *p < 0.05. **(B)** The relative expression of the CBXs family members in OV.

First, we analyzed mRNA expression and protein expression of different CBXs family to explore their potential prognostic and therapeutic value in ovarian cancer patients. As shown in Supplementary Figure S1 and Supplementary Table S3, mRNA expression of CBXs members in various cancer types was explored by using the ONCOMINE database. Significant down-regulation of CBX1 and CBX7 was found in ovarian cancer (OV) tissues compared to normal tissues, while the expression levels of CBX3 were significantly up-regulated in OV tissues. In Bonome Ovarian dataset (Bonome et al., 2008), 2.56-fold decrease in CBX1 expression was shown in OV tissues (p = 3.91E-12). Similarly, the result from three datasets including Bonome Ovarian dataset, TCGA dataset, Yoshihara Ovarian dataset (Yoshihara et al., 2009) showed that there were 2.937-fold (p = 5.43E-12), 3.739-fold (p = 4.50E-6), and 5.422-fold (p = 7.48E-10) decrease in CBX7 mRNA expression in OV tissues compared to normal tissues, respectively. However, in the TCGA dataset, CBX3 overexpression was shown in OV tissues with a fold change of 2.064 (p = 1.40E-7). Furthermore, the mRNA expression levels of CBXs family were further explored by the GEPIA2 whose resources were different from ONCOMINE database. As shown in Figure 1A, the result supported that the mRNA expression of CBX7 was decreased in OV tissues compared with normal tissues. Besides, significant down-regulation of CBX6 and up-regulation of CBX8 were further obtained in OV tissues compared to normal tissues (Figure 1A). Then, we compared the relative expression of distinct CBXs members in OV by GEPIA2, and the result showed that CBX3 had the highest relative expression levels among all eight CBXs family members (Figure 1B).

**FIGURE 2:**
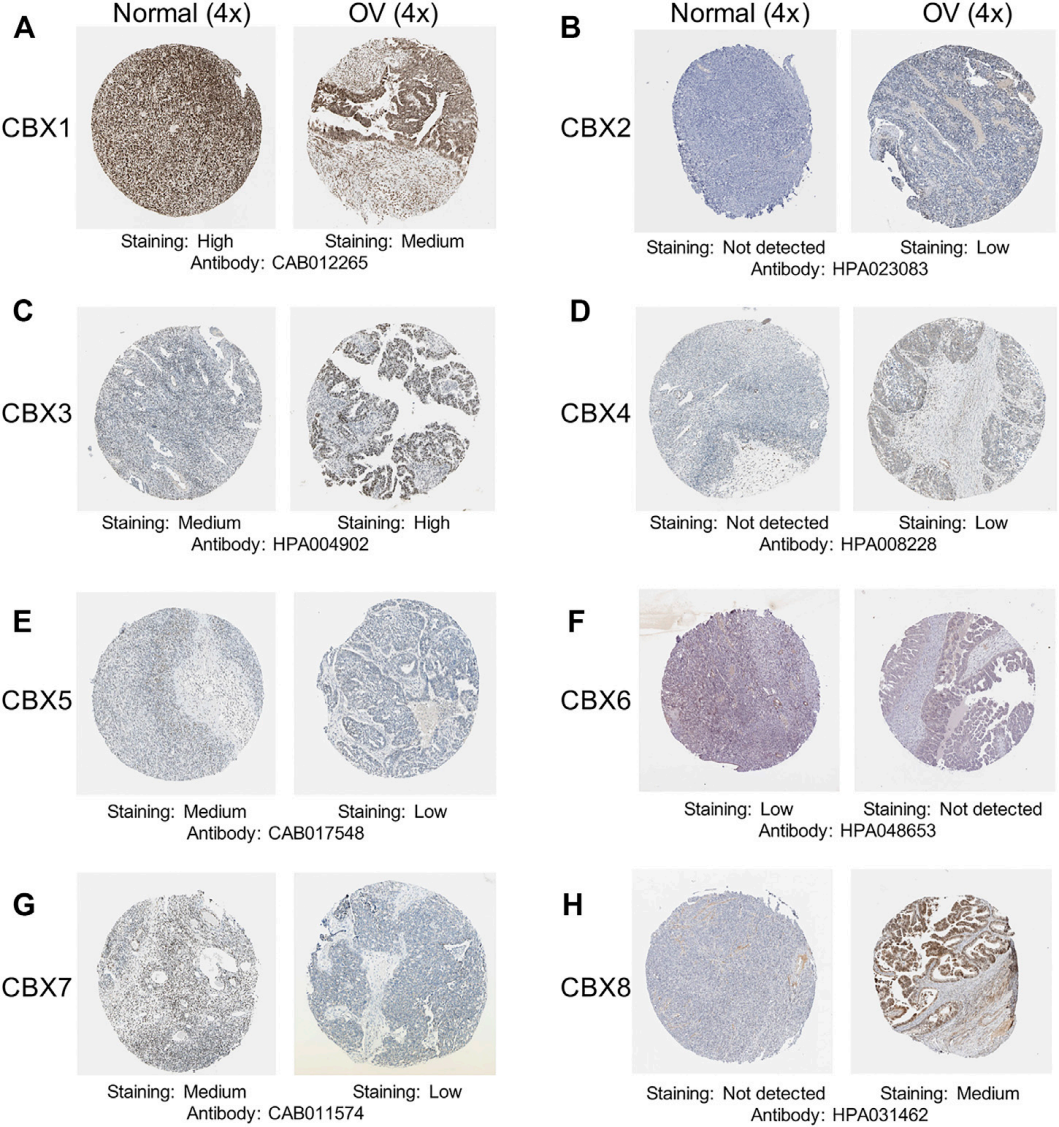
Representative immunohistochemistry images of the CBXs family members in OV tissues and normal ovarian tissues. **(A–H)** The protein expression profiles of CBX1-8 were collected from the Human Protein Atlas database.

After a comprehensive analysis of the CBXs mRNA expression pattern in OV, we tried to use the Human Protein Atlas to investigate the protein expression levels of CBXs family in OV. As shown in Figure 2, the protein expression levels of CBX1/6/7 were decreased in OV tissues compared with normal ovarian tissues (high vs. medium; low vs. not detected; medium vs. low, respectively) (Figures 2A, F,G). Meanwhile, higher protein expression levels of CBX3/8 were found in OV tissues compared to normal tissues (medium vs. high; not detected vs. medium, respectively) (Figures 2C,H). These findings were consistent with our previous findings on the mRNA expression of CBXs family. Moreover, CBX2/4 had not detected expression in normal ovarian tissues and low expression in OV tissues (Figures 2B,D), while CBX5 had medium expression in normal tissues and low expression in OV tissues (Figure 2E).

**FIGURE 3:**
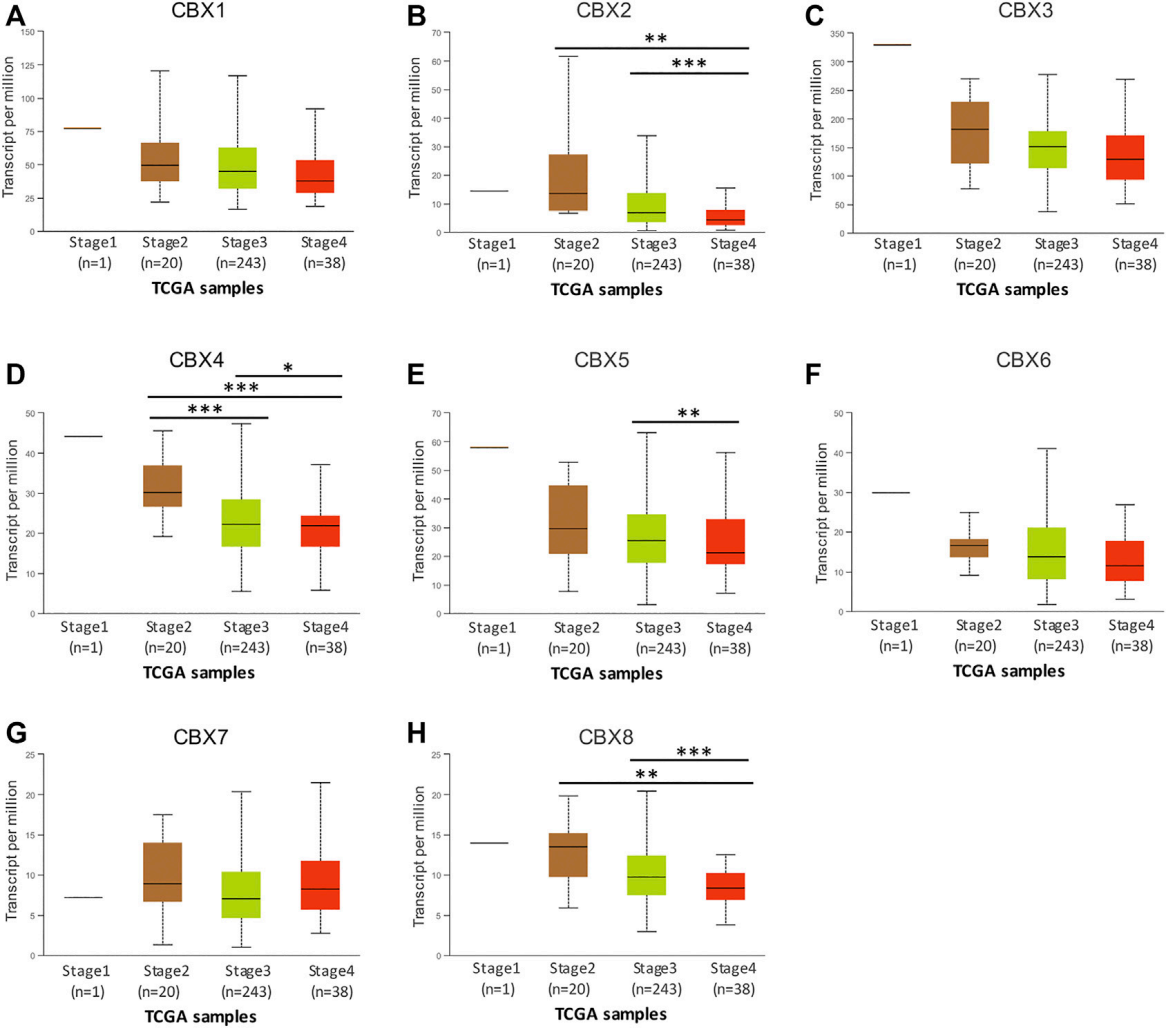
The relationship between the mRNA expression levels of different CBXs members and individual cancer stages in patients with OV. **(A–H)** The relationship information of CBX1-8 was evaluated from the UALCAN database. *p < 0.05, **p < 0.01, ***p < 0.001.

Next, we explored the relationship between the mRNA expression of eight CBXs members and the individual cancer stages of OV by using the UALCAN database. The results showed that the mRNA expression levels of CBX2/4/5/8 were significantly correlated with individual cancer stages of OV, and patients who had higher cancer stages tended to express lower mRNA expression levels of CBXs. The lowest mRNA expression levels of CBX2/4/5/8 were detected in cancer stage 4 (Figures 3B,D,E,H). Moreover, the mRNA expression levels of CBX1/3/6 had a trend to lower expression in more advanced cancer stages, although that was not statistically significant (Figures 3A,C,F,G). That may be due to the small sample size and other reasons.

### Prognostic Value of mRNA Expression of the CBXs Family in Ovarian Cancer Patients

**FIGURE 4:**
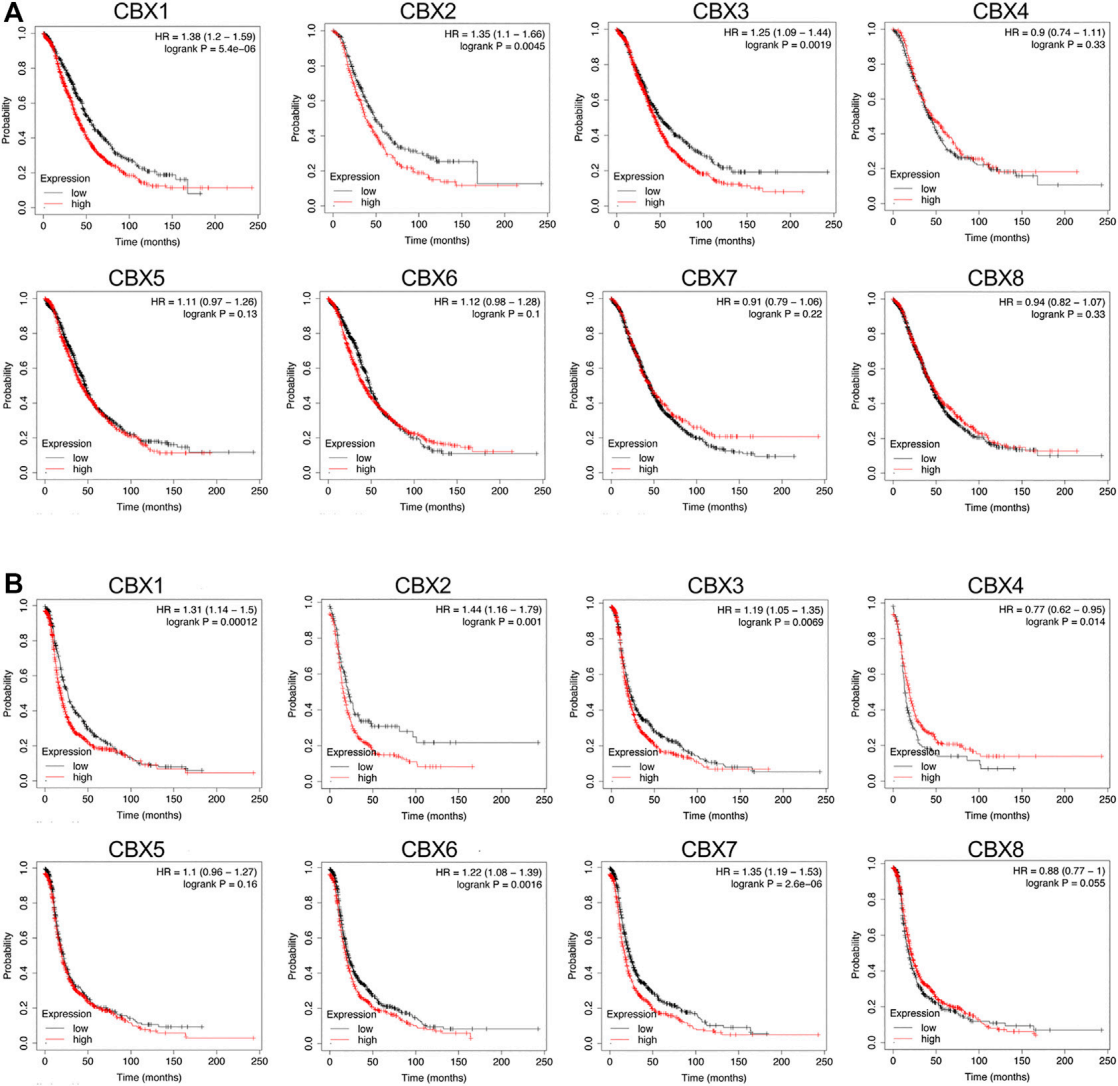
Prognostic value of the mRNA expression levels of the CBXs family members in patients with OV (the Kaplan-Meier Plotter). **(A)** The overall survival (OS) curve of CBX1-8 in patients with OV. **(B)** The progression-free survival (PFS) curve of CBX1-8 in patients with OV.

Further, we applied the Kaplan–Meier plotter database to investigate the prognostic value of mRNA expression of all eight CBXs members in patients with OV. As shown in Figure 4, higher mRNA expression levels of CBX1 (OS: HR = 1.38 (1.20–1.59), *p* < 0.001; PFS: HR = 1.31 (1.14–1.5), *p* = 0.00012), CBX2 (OS: HR = 1.35 (1.1–1.66), *p* = 0.0045; PFS: HR = 1.44 (1.16–1.79), *p* = 0.001) and CBX3 (OS: HR = 1.25 (1.09–1.44), *p* = 0.0019; PFS: HR = 1.19 (1.05–1.35), *p* = 0.0069) were all significantly associated with shorter overall survival (OS) and progression-free survival (PFS) for OV patients (Figure 4).

In contrast to the survival analysis results for CBX1/2/3 in OV patients, survival analysis focusing on the other five CBXs family members showed either irrelevant or inconsistent results for both OS and PFS in OV patients. Higher mRNA expression of CBX4 was not related to OS [HR = 0.9 (0.74–1.11), *p* = 0.33] but was related to favorable PFS [HR = 0.77 (0.62–0.95), *p* = 0.014] of OV patients. In addition, increased expression of CBX6 [HR = 1.12 (0.98–1.28), *p* = 0.1] and CBX7 [HR = 0.91 (0.79–1.06), *p* = 0.22] was not related to OS of OV patients, while increased expression of CBX6 [HR = 1.22 (1.08–1.39), *p* = 0.0016] and CBX7 [HR = 1.35 (1.19–1.53), *p* < 0.001] was significantly correlated with poor PFS of OV patients. Finally, the mRNA expression of CBX5 [OS: HR = 1.11 (0.97–1.26], *p* = 0.13; [PFS: HR = 1.1 (0.96–1.27), *p* = 0.16] and CBX8 [OS: HR = 0.94 (0.82–1.07), *p* = 0.33; PFS: HR = 0.88 (0.77–1), *p* = 0.055] did not show any relation to the prognosis in OV patients (Figure 4).

### CBX3 Promoted Ovarian Cancer Cell Proliferation and Impacts the Treatment Outcomes of Ovarian Cancer Patients

**FIGURE 5:**
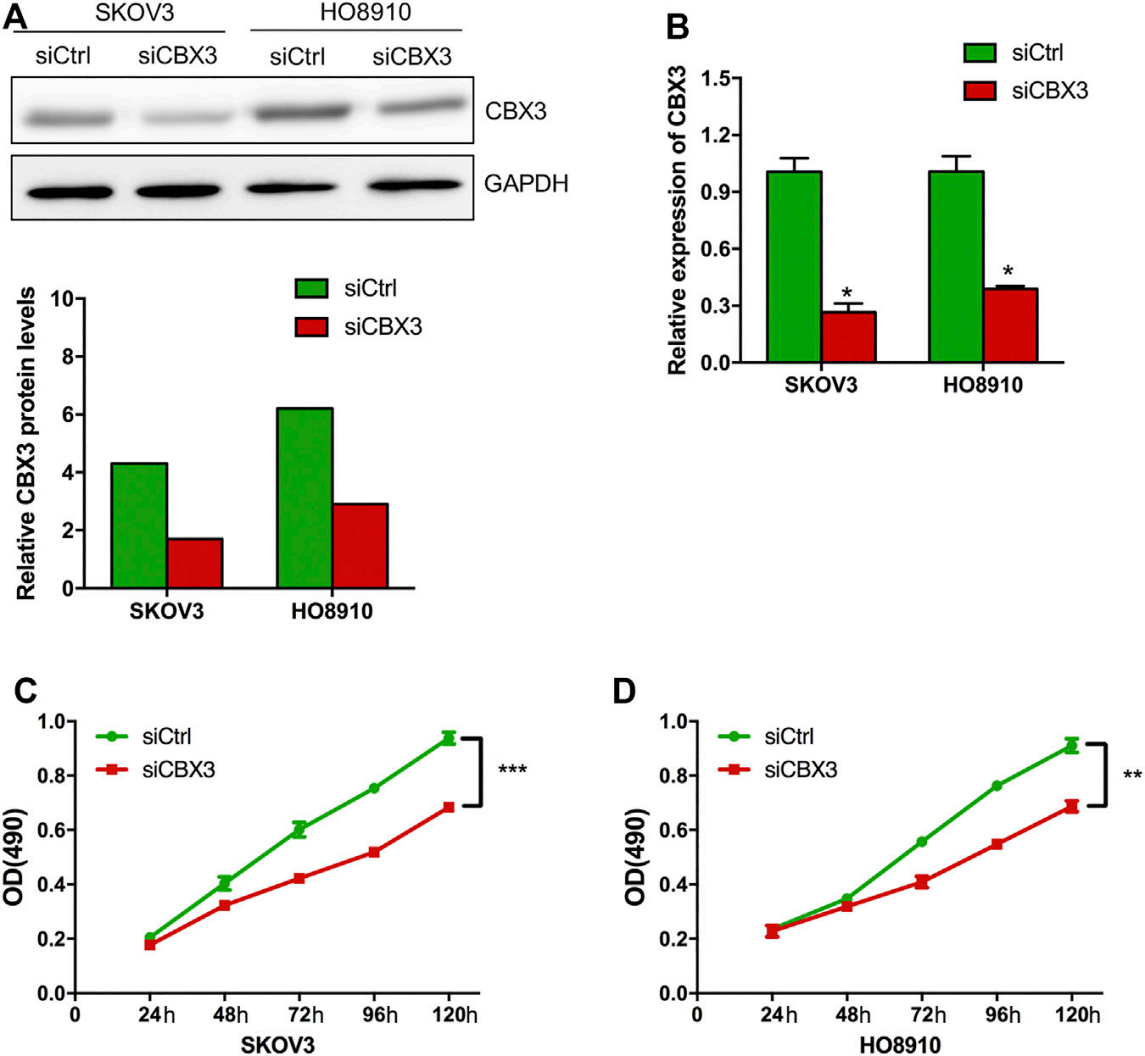
CBX3 knockdown inhibited the proliferation of ovarian cancer cells. **(A)** The knockdown efficiency of CBX3 in SKOV3 and HO8910 was evaluated by Western Blot. **(B)** The knockdown efficiency of CBX3 in SKOV3 and HO8910 was assessed by qRT-PCR. **(C–D)** CCK-8 assay was conducted to evaluate the effect of siCBX3 on the proliferation of SKOV3 and HO8910 cell lines. **p < 0.01, ***p < 0.001.

Based on the above expression and prognosis results of CBX family members, we found that only CBX3 was overexpressed in ovarian cancer tissues and closely related to the poor prognosis (OS and PFS) of patients with OV at the same time, indicating that CBX3 is the most likely potential diagnostic indicator and new therapeutic target for patients with ovarian cancer. Thus, we further investigated the function of CBX3 in ovarian cancer cells. CBX3 was reported to play an important role in cell growth in some cancer types, however, it is role in OV is still unclear. To investigate the impact of CBX3 on the proliferation of ovarian cancer cells, we conducted CCK8 assay to analyze the growth of ovarian cancer cells which were transfected with CBX3 siRNA (siCBX3) or siRNA control (siCtrl). The knockdown efficiency was evaluated and the results showed that compared with siCtrl group of two ovarian cancer cell lines including SKOV3 and HO8910, the expression of CBX3 was decreased in siCBX3 group in both protein and mRNA levels (Figures 5A,B). Next, we conducted CCK8 assay and the results showed that compared with siCtrl group, the growth of ovarian cancer cells was inhibited after transfected with siCBX3 (Figures 5C,D), suggesting that CBX3 was involved in the proliferation of ovarian cancer cells.

**FIGURE 6:**
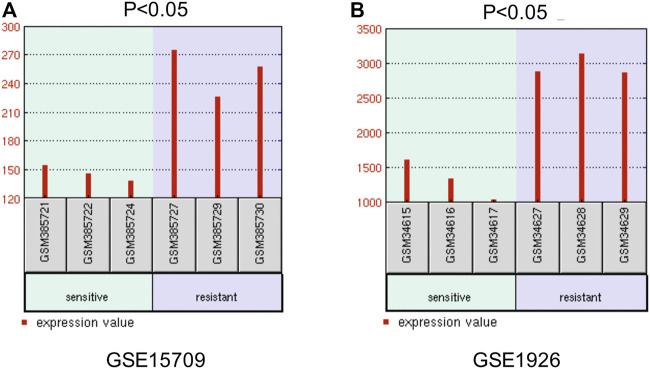
The effect of CBX3 on the treatment outcomes of ovarian cancer patients. **(A–B)** GSE15709 and GSE1926 are two datasets related to platinum-based chemotherapy and are used to explore the impacts of CBX3 expression levels on ovarian cancer therapy.

Furthermore, we explored the effect of CBX3 on the treatment outcomes of ovarian cancer patients. The expression level of CBX3 was checked in two microarray datasets related to platinum chemotherapy including GSE15709 and GSE1926. The results from these two datasets both presented that treatment with the platinum drugs upregulated the expression level of CBX3 expression in ovarian cancer tissues or cells (Figure 6). These results indicated that CBX3 may influence the chemotherapy responses of ovarian cancer patients.

### Genetic Alteration and Functional Analysis of the CBXs Family in Ovarian Cancer Patients

**FIGURE 7:**
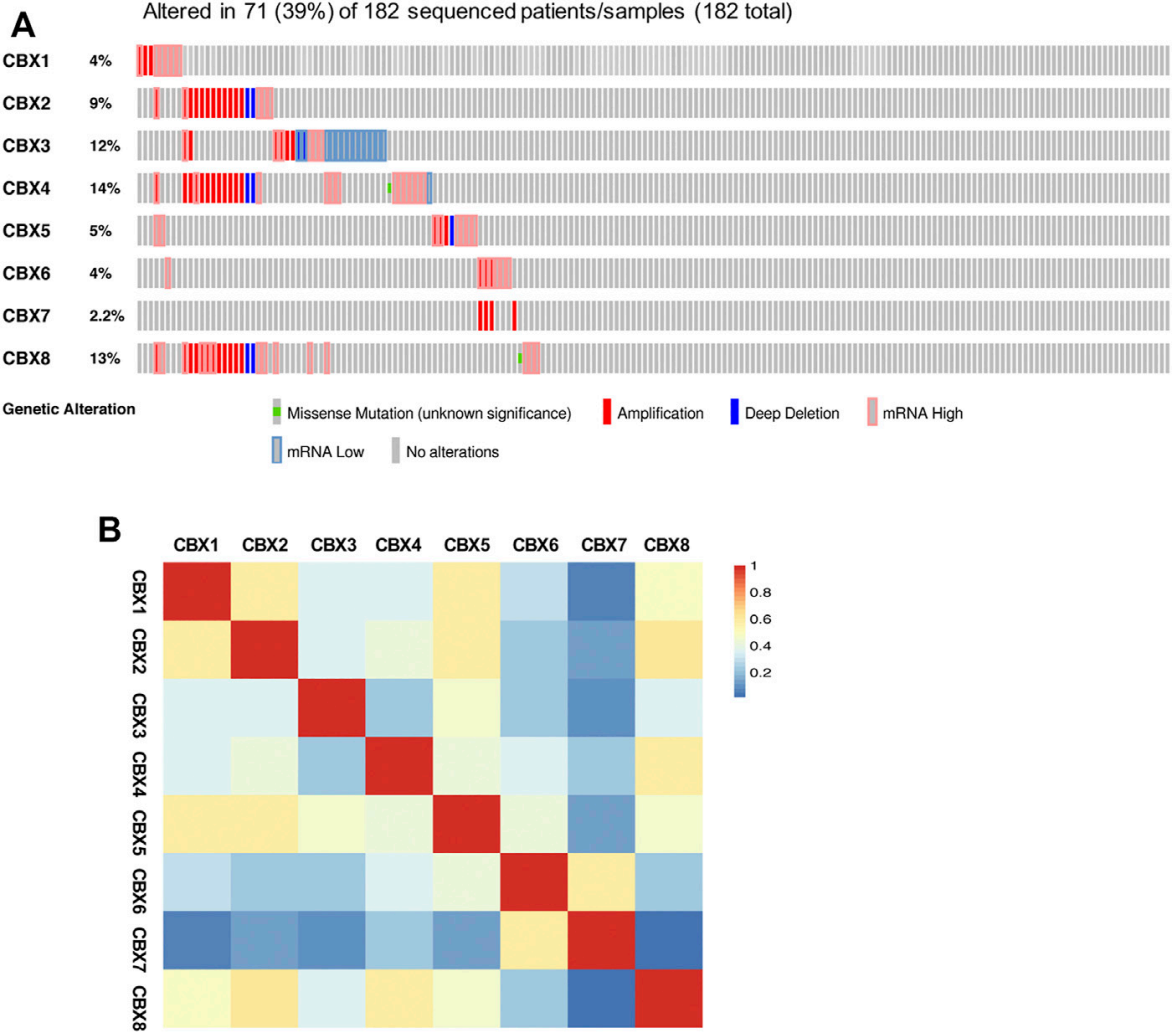
Genetic alterations and correlation analysis of distinct CBXs members in OV. **(A)** Summary of the alteration rates for CBX1-8 in OV (cBioPortal). **(B)** Correlation between eight CBXs family members in OV (GEPIA2).

Next, we explored the genetic alterations of individual CBXs family members by using the temporary TCGA dataset. As shown in Figure 7A, all eight CBXs family members were altered in OV patients, with 4, 9, 12, 14, 5, 4, 2.2, and 13% alteration rates, respectively. CBX4/8/3 ranked the highest three genes with the genetic alteration. In the 182 sequenced OV patients/samples, the genetic alteration was detected in 71 cases and the total alteration rate was 39% (Figure 7A). Then, we measured the correlation between each CBXs member at the transcriptional level by using the GEPIA2, and Pearson’s correction was included. A remarkably positive correlation between CBX2 and CBX8, CBX1 and CBX5 were observed (Figure 7B).

**FIGURE 8:**
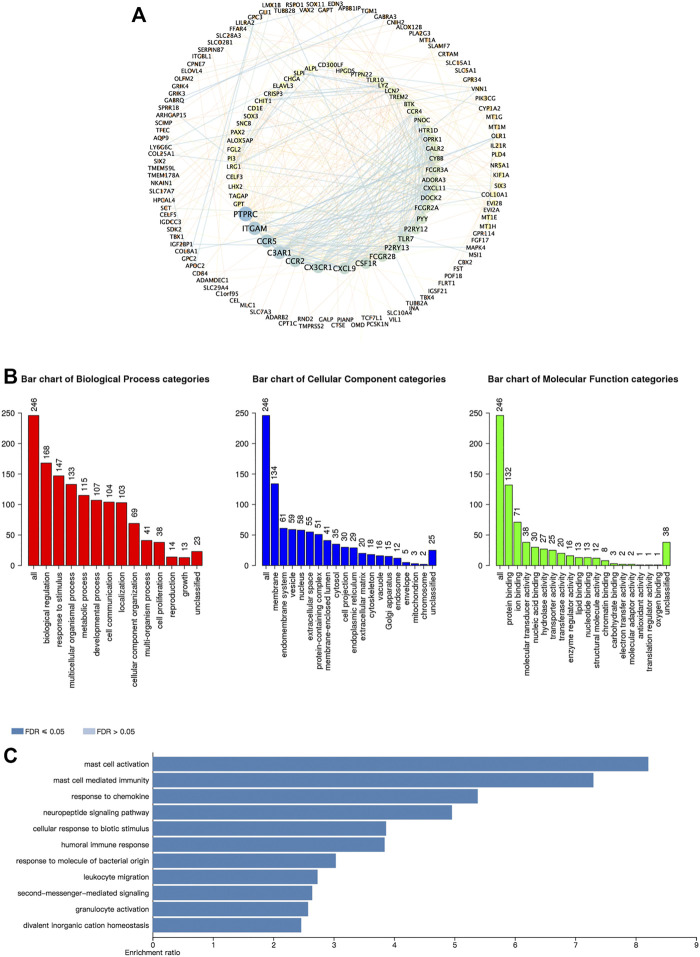
Predicted functions and pathways of the CBXs family members and their 246 co-expressed molecules in OV. **(A)** PPI network of the CBXs family interaction partners was constructed (cBioPortal and Cytoscape). The colors of nodes and edges: high values to dark colors; low values to bright colors. **(B)** Biological process, cellular components, and molecular functions of the CBXs family interaction partners was predicted by GO functional enrichment analysis (WebGestalt). **(C)** KEGG pathway analysis on the CBXs family interaction partners was shown (WebGestalt).

We further investigated the function of the CBXs Family in OV patients. Since lots of proteins synergistically performed their functions by forming protein-protein complexes, it is essential to discover protein-protein interaction (PPI) patterns for better understanding the function of the CBXs family. We analyzed 246 frequently altered neighbor genes that were most relevant with CBXs members in OV patients by the cBioPortal and constructed an integrated network using the Cytoscape (Figure 7A, Supplementary Table S2). The results showed that PTPRC, ITGAM, CCR5, C3AR1, and CCR2 were primarily related to the function of the CBXs family in OV (Figure 8A). In addition, the biological functions of CBXs members and their 246 co-expressed genes were analyzed by GO annotation and KEGG pathway analysis using the WebGestalt (Figures 8B,C). Biological processes including biological regulation, response to stimulus, and multicellular organismal process were significantly regulated by the CBXs family. Cellular components such as membrane, endomembrane system and vesicle were significantly associated with the function of CBXs family. Besides, the CBXs family mainly affected the molecular functions including protein binding, ion binding, and molecular transducer activity (Figure 8B). As shown in Figure 8C, in terms of KEGG pathway analysis, 11 pathways were related to the function of CBXs family in OV, and the top three most relevant pathways were mast cell activity, mast cell-mediated immunity, and response to a chemokine (Figure 8C).

### Immune Infiltration Levels of the CBXs Family in Ovarian Cancer Patients

**FIGURE 9:**
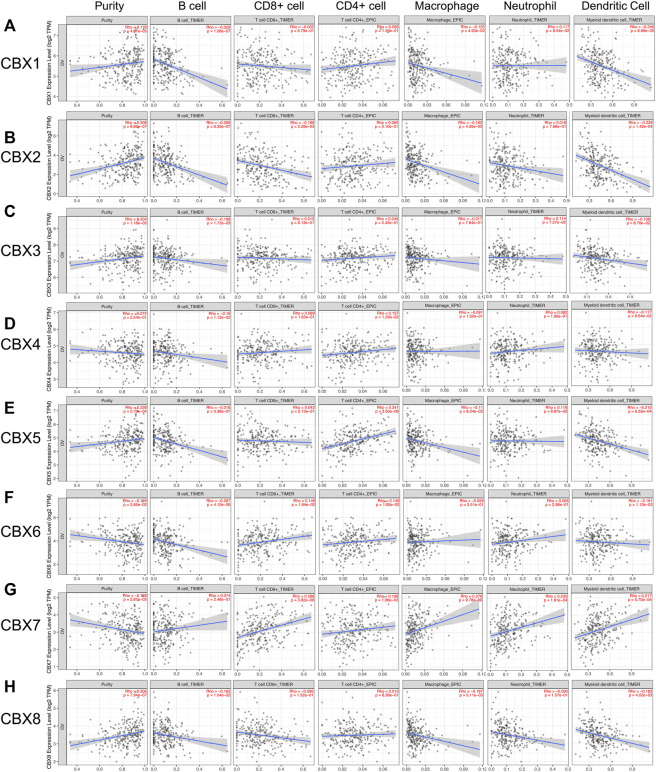
The correlations between differentially expressed CBXs family members and immune cell infiltration. **(A–H)** The effects of CBX1-8 on the immune cell infiltration were analyzed by TIMER2.0. Six immune cells were analyzed including B cells, CD8^+^ T cells, CD4^+^ T cells, macrophages, neutrophils, and dendritic cells.

The CBXs family were reported to participate in the tumor progression and the several immune cells infiltration in various cancer types, thus affecting patient prognosis and therapy (Chen et al., 2019; Zhou et al., 2021). In this study, we applied TIMER2.0 to explore the correlation between the individual CBXs family members and the immune infiltration of ovarian cancer. As shown in Figures 9A,B,H, CBX1/2/8 were all negatively correlated with B cells, macrophages, and dendritic cells, CBX2 was also negatively correlated with CD8 + T cells. CBX3 was negatively correlated with B cells (Figure 9C). CBX4/5 were negatively correlated with B cells and positively correlated with CD4 + T cells, CBX5 was also negatively correlated with dendritic cells (Figures 9D,E). CBX6 was negatively correlated with B cells and dendritic cells and positively correlated with CD8 + T cells and CD4 + T cells (Figure 9F). In addition, there were positive correlations between CBX7 and the infiltration of five immune cells including CD8 + T cells, CD4 + T cells, macrophages, neutrophils, and dendritic cells (Figure 9G, Supplementary Figure S2).

### Methylation Expression Levels of the CBXs Family in Ovarian Cancer Patients

Increasing reports have indicated that DNA methylation is negatively associated with gene expression in cancers (Győrffy et al., 2016). Thus, in the present study, we tried to analyze whether the methylation status was correlated with the expression of CBXs members. We obtained the methylation expression data of CBXs family by using the DiseaseMeth. As shown in Supplementary Figure S3, the methylation expression levels of CBX2, CBX3, CBX4, CBX5, and CBX8 were significantly decreased in OV tissues compared to normal tissues. Our previous results on mRNA expression of CBXs members indicated that the upregulated expression of CBX3 and CBX8 in OV patients, these aberrant expression levels of CBX3/8 might be due to their low methylation levels in OV tissues. For the rest of CBXs members, in addition to methylation, there may be other factors affecting their expression levels, such as gene mutation, fusion, epigenetic modification, and so on.

## Discussion

Numerous studies reported that except for tumor genetics, abnormal epigenetic regulation has also been depicted to play a critical role in the development of ovarian cancer (Matei and Nephew, 2020). The CBXs family members, as critical components of epigenetic regulation complexes, are reported to take part in the development and progression of various cancer types, including ovarian cancer (Li et al., 2014a; Li et al., 2020c). Some researchers found that high expression of CBX1 was significantly related to an unfavorable progression and was correlated with chemoresistance in breast cancer (Liang et al., 2017). In hepatocellular carcinoma (HCC), CBX2 was found to promote HCC cells proliferation by decreasing YAP phosphorylation (Mao et al., 2019). High expression of CBX3 was observed in several cancers such as HCC and glioma and could accelerate cell proliferation (Zhao et al., 2019; Zhong et al., 2019). Hu et al. reported that knockdown of CBX4 could inhibit cell growth and migration in lung cancer through regulating the BMI-1 pathway (Hu et al., 2020). High expression of CBX5 was detected in gastric cancer and can promote cell migration and invasion (Guo et al., 2018). Zheng et al. reported that a higher expression level of CBX6 was associated with a worse prognosis in patients with HCC (Zheng et al., 2017). In our previous studies, CBX7 was proved to make TWIST-1 transcriptionally nonfunctional during mesenchymal-epithelial transition (MET) process in ovarian cancer, and a subclassification of ovarian cancers based on both CBX7 and TWIST-1 expression was then described which could predict clinical outcomes and patient prognosis in OV (Li et al., 2020c). Finally, CBX8 was found to promote invasion and migration in several cancer types including glioblastoma, lung cancer, and breast cancer (Jia et al., 2020). Despite a few members of the CBXs family having been discovered to play important roles in tumors, distinct roles of the CBXs family in ovarian cancer remained to be illuminated. In this work, the expression, gene alteration, prognostic values, immune infiltration, and methylation of different CBXs members in ovarian cancer were analyzed.

For the first time, the mRNA expression levels of eight CBXs family members were analyzed and summarized in ovarian cancer tissues compared with normal ovarian tissues by using both the ONCOMINE database and the GEPIA2 database. Higher mRNA expression levels of CBX3/8 and lower expression levels of CBX1/6/7 were observed in OV tissues compared to normal tissues. These mRNA expression data were consistent with the subsequent protein data of CBXs family. In addition, a further novel finding was that despite the mRNA expression levels of CBX2/4/5 were not statistically different between OV tissues and normal tissues, their mRNA expression levels in ovarian cancer tissues were significantly correlated with individual cancer stages. Ovarian cancer patients who had higher cancer stages tended to express lower mRNA expression levels of these CBXs. Besides, the mRNA expression levels of CBX1/3/6 showed a significant difference in OV tissues compared to normal tissues, however, their expression levels only had a trend to lower expression in more advanced cancer stages but without statistical significance. That may be due to the small sample size and other reasons.

Nowadays, the prognostic value of several CBXs members in cancers has been reported. CBX1 has been detected to have high protein expression in HCC which indicated that HCC patients with high expression of CBX1 had worse clinical outcomes (Yang et al., 2018). Elevated protein expression of CBX3 was involved in unfavorable prognosis in prostate cancer, and it also was an independent prognostic marker (Slezak et al., 2013). In lung cancer, CBX3 mRNA expression was increased and was associated with a shorter survival time (Alam et al., 2018). However, their prognostic values in ovarian cancer have not been fully illuminated. In this analysis, we explored the prognostic roles of eight CBXs family members in OV patients by using the Kaplan–Meier plotter. The results showed that CBX1, CBX2, and CBX3 were all associated with worse OS and PFS in OV patients. Nevertheless, the prognostic value of the other five CBXs family members for predicting OS and PFS in ovarian cancer patients was either irrelevant (CBX5 and CBX8) or inconsistent (CBX4, CBX6, and CBX7). These results suggested that more research was needed to further investigate their prognostic significance. According to these findings of the expression and prognosis of CBXs, we found that only CBX3 had consistent results in expression and prognosis. Thus, further cell experiments were conducted to explore the function of CBX3 in OV, and the results showed that CBX3 could promote ovarian cancer cell proliferation and impact the treatment outcomes of OV patients. These findings suggested that CBX3 may be the potential diagnostic indicator and new therapeutic target in ovarian cancer.

It is widely known that genetic alteration is a general phenomenon in various tumors including ovarian cancer and plays a critical role in several cell biological processes such as cell growth, cell apoptosis, and cell cycle (Sanchez-Vega et al., 2018). All eight CBXs family members were detected to be altered in OV patients, and the total genetic alteration rate of CBXs family was 39%. The results above comprehensively suggested that genetic alterations of the CBXs family probably play a pivotal role in ovarian cancer. Moreover, methylation is also associated with the expression levels of genes and takes part in tumor development (Győrffy et al., 2016). There were several clues that existed revealing the role of methylation of CBXs in cancers. For example, CBX1, CBX3, and CBX5 were found to be act as the methyl readers which were involved in the interpretation of H3K9me3 marks induced by H3K9 methyltransferases (van Wijnen et al., 2021). Moreover, differentially expressed CBXs were found to have a strong association with the SUMOylation of DNA methylation proteins in colorectal cancer (Li et al., 2020d). In our study, we showed that higher expression levels of CBX3/8 in OV tissues might be due to their low methylation levels. For the rest of CBXs members, in addition to methylation, there may be other factors affecting their expression levels, such as genetic alterations that we mentioned above.

Next, the molecular biological functions of CBXs members and their 246 co-expressed genes were analyzed. Protein-protein network interactions exhibited PTPRC, ITGAM, and CCR5 were most related to the modulation and function of the differentially expressed CBXs family members in ovarian cancer. Biological processes such as mast cell activation, mast cell-mediated immunity were remarkably regulated by the CBXs family members in ovarian cancer. Mast cells are a kind of immune cells that could secrete diverse active compounds to take part in the immune response (Komi and Redegeld, 2020). Giuseppe et al. reported that mast cell density was increased in gastric cancer and exerted a protumorigenic role through the release of angiogenic and lymphangiogenic factors (Sammarco et al., 2019). Mast cell infiltration was found to participate in poor response to neoadjuvant chemotherapy in inflammatory breast cancer (Reddy et al., 2019). Despite mounting research showing that mast cells were consistently infiltrating tumors, the consequences of their presence as tumor suppressors or tumor drivers still remain unclear (Aponte-López et al., 2018). Particularly, in ovarian cancer, their roles and functions were still under discussion. Our results suggested that CBXs family may play a critical role in mast cell infiltration in ovarian cancer.

Mounting evidence supported that immune cell infiltration was significantly associated with tumor progression and recurrence and is considered as a pivotal determinant of clinical outcome and immune therapy response (Li et al., 2016). Lele et al. showed that tumor-infiltrating immune cells were correlated with clinical features in colorectal cancer and could act as a prognostic marker (Ye et al., 2019). Liu et al. found that profiling of immune infiltration plays an important role in the prediction of prognosis in ovarian cancer, and it could also be considered in therapeutic modulation (Liu et al., 2020). Moreover, epigenetic regulation of innate immune response is an emerging field these years. There were evidence showing that in macrophages, CBX2 can bind to and recruit Jmjd3 to the Ifnb promoter, resulting in the demethylation of H3K27me3, thereby increasing the transcription of IFN-β, an essential factor involved in the antiviral innate immunity (Sun et al., 2019). In addition, another published paper also demonstrated that CBX7 knockdown leads to apoptosis of CD4^+^ T cells *via* hyper demethylation of FasL gene promoter and increased expression of FasL (Li et al., 2014b). In this work, we found a remarkable correlation between the expression of individual CBXs family members and the infiltration of six immune cells, thus suggesting that CBXs members may reflect the immune status of ovarian cancer. Particularly, we found that almost all CBXs members except CBX7 were significate associated with B cell infiltration in ovarian cancer. B cell, as a critical immune cell, harbored various functions in the immune response. Increasing studies supported that tumor-infiltrating B lymphocytes could suppress tumor progression by secreting antibodies, promoting T cell response, and killing tumor cells directly (Wang et al., 2019). Several studies reported that B cell infiltration was related to poorer survival in patients with ovarian cancer (Dong et al., 2006; Yang et al., 2013). Furthermore, in the present study, we found that CBX7 was positive correlations with five of six immune cells infiltration including CD8 + T cells, CD4 + T cells, macrophages, neutrophils, and dendritic cells, suggesting that CBX7 may play a critical role in affecting the immune status of ovarian cancer.

There were several limitations in this work. Firstly, most of the data used for analysis in this study were retrieved from online services, further cell-based studies and clinical experiments were needed to confirm our results and to investigate the clinical application of the CBXs family in ovarian cancer. Besides, we lacked the research on the detailed mechanisms of individual CBXs members in ovarian cancer. Further studies were required to explore the specific mechanism between each CBXs member and ovarian cancer.

## Conclusion

In conclusion, we first comprehensively analyzed the expression, prognostic values, gene alteration, immune infiltration, and methylation of different CBXs family members in ovarian cancer. Higher expression of CBX3/8 and lower expression of CBX1/6/7 were detected in OV tissues. CBX1/2/3 were all associated with worse OS and PFS in OV patients. CBX3 promoted ovarian cancer cell proliferation and impacts the treatment outcomes of OV patients. Besides, a high genetic alteration rate of CBXs family (39%) was observed in OV. The low methylation status of CBX3/8 may be associated with their high expression levels in OV. Moreover, individual CBXs members were associated with varying degrees of the infiltration of immune cells, especially B cells. These findings exhibited the pivotal value of the CBXs family in the prognosis and therapy of ovarian cancer. Our results may provide new insight to explore new prognostic biomarkers and therapeutic targets for ovarian cancer.

## Data Availability

The datasets presented in this study can be found in online repositories. The names of the repository/repositories and accession number(s) can be found in the article/Supplementary Material.

## Supplementary Material

Supplementary Figure S1 Transcriptional expression levels of the CBXs family members in different cancer types (ONCOMINE). Red cell represented gene overexpression; blue cell represented decreased expression. The numbers in each cell represented the evidential frequencies. The deeper the color, the higher the significance.

Supplementary Figure S2 The heat map for the correlations between differentially expressed CBXs family members and immune cell infiltration.

Supplementary Figure S3 The methylation levels of distinct CBXs family in OV. **(A–H)** The methylation values of CBX1-8 were collected from the DiseaseMeth database.
