# Supplementary material for: Prognostic Value and Therapeutic Potential of CBX Family Members in Ovarian Cancer
Source: Front Cell Dev Biol. 2022 Jan 27;10:832354. doi: 10.3389/fcell.2022.832354 (PMC8829121; doi:10.3389/fcell.2022.832354)
Supplement: Supplementary file 1 [file Table1.DOCX]

| **Databases** | **Authors** | **Samples** | **Homepage links** |
| --- | --- | --- | --- |
| Oncomine | Rhodes DR. et al. | Tissues | http://www.oncomine.org |
| GEPIA2 | Tang Z. et al. | Tissues | http://gepia.cancer-pku.cn/ |
| The Human Protein Atlas | [Anna Asplund](https://pubmed.ncbi.nlm.nih.gov/?sort=date&term=Asplund+A&cauthor_id=22623277). et al. | Tissues | https://www.proteinatlas.org/ |
| UALCAN | Chandrashekar DS | Tissues | http://ualcan.path.uab.edu/index.html |
| Kaplan-Meier plotter | Gyorffy B. et al. | Tissues | http://kmplot.com/analysis/ |
| cBioPortal | Cerami E. et al. | Tissues | http://www.cbioportal.org/ |
| WebGestalt | Liao Y. et al. | - | http://webgestalt.org/ |
| Cytoscape | Doncheva NT et al. | - | - |
| TIMER2.0 | Li T. et al. | Tissues | https://cistrome.shinyapps.io/timer/ |
| DiseaseMeth 2.0 | Xiong Y. et al. | Tissues | http://bio-bigdata.hrbmu.edu.cn/diseasemeth/ |

**Supplementary Table S1.** A series of bioinformatics databases for analyzing the role of the CBXs family members in ovarian cancer.
