# Supplementary figures and images for: Prognostic Value and Therapeutic Potential of CBX Family Members in Ovarian Cancer
Source: Front Cell Dev Biol. 2022 Jan 27;10:832354. doi: 10.3389/fcell.2022.832354 (PMC8829121; doi:10.3389/fcell.2022.832354)

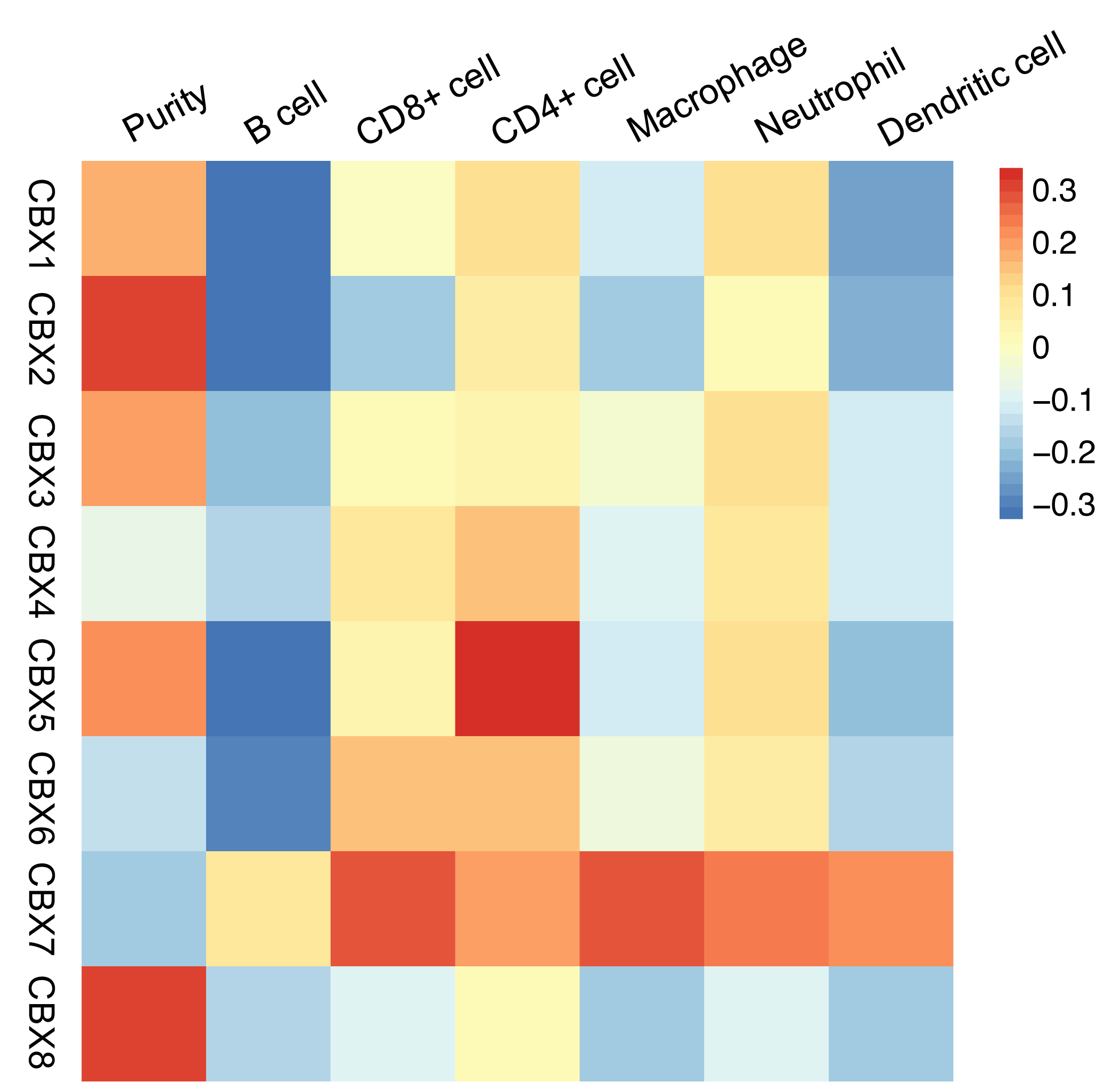

Supplement: Supplementary file 2 [file Image3.TIF]

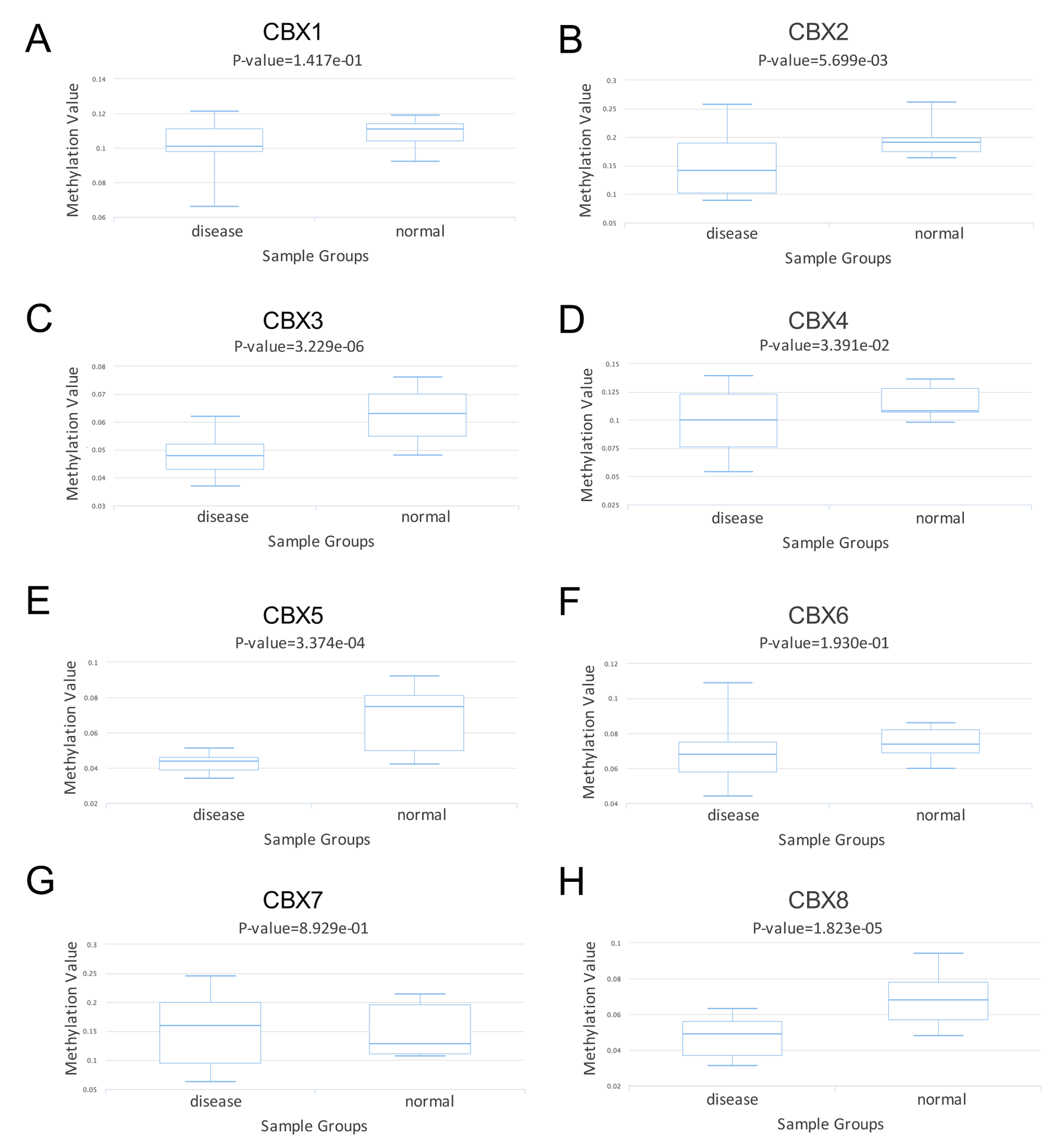

Supplement: Supplementary file 3 [file Image2.TIF]

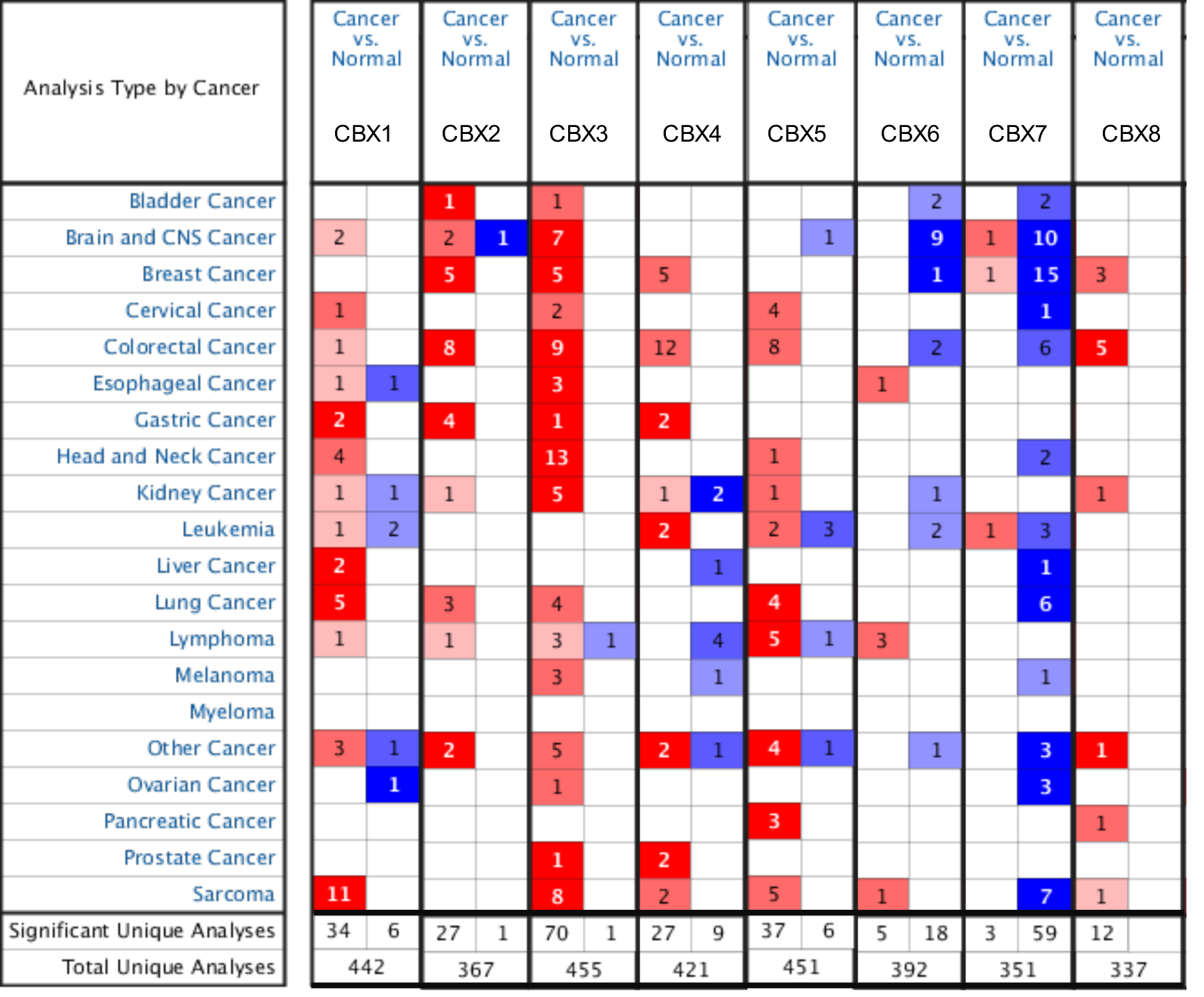

Supplement: Supplementary file 4 [file Image1.TIF]
