# Supplementary material for: Prognostic Value and Therapeutic Potential of CBX Family Members in Ovarian Cancer
Source: Front Cell Dev Biol. 2022 Jan 27;10:832354. doi: 10.3389/fcell.2022.832354 (PMC8829121; doi:10.3389/fcell.2022.832354)
Supplement: Supplementary file 5 [file Table2.DOCX]

**Supplementary Table S2:** The CBXs family members associated co-expressed molecules in ovarian cancer.

| Gene | Log Ratio | p-Value | expression |
| --- | --- | --- | --- |
| P2RY12 | -1.15 | 1.65E-06 | Unaltered group |
| FFAR4 | -1.05 | 2.35E-06 | Unaltered group |
| HTR1D | 0.92 | 1.20E-05 | Altered group |
| GPR34 | -0.86 | 1.41E-05 | Unaltered group |
| ADORA3 | -0.83 | 1.45E-05 | Unaltered group |
| CBX2 | 0.88 | 1.83E-05 | Altered group |
| H2BFXP | 0.89 | 2.02E-05 | Altered group |
| P2RY13 | -0.97 | 2.76E-05 | Unaltered group |
| GAPT | -0.99 | 3.09E-05 | Unaltered group |
| CX3CR1 | -1 | 4.00E-05 | Unaltered group |
| TLR10 | -0.92 | 5.12E-05 | Unaltered group |
| MT1G | -1.54 | 5.55E-05 | Unaltered group |
| SCIMP | -0.72 | 5.72E-05 | Unaltered group |
| EVI2B | -0.79 | 7.37E-05 | Unaltered group |
| AADAT | 0.73 | 7.77E-05 | Altered group |
| ALOX5AP | -0.96 | 7.88E-05 | Unaltered group |
| TLR7 | -0.85 | 8.72E-05 | Unaltered group |
| CXORF21 | -0.74 | 8.79E-05 | Unaltered group |
| PIK3CG | -0.8 | 1.07E-04 | Unaltered group |
| APBB1IP | -0.83 | 1.08E-04 | Unaltered group |
| CRTAM | -0.89 | 1.26E-04 | Unaltered group |
| SRSF12 | 0.76 | 1.39E-04 | Altered group |
| TREM2 | -0.81 | 1.49E-04 | Unaltered group |
| SIRPB2 | -0.82 | 1.69E-04 | Unaltered group |
| MT1DP | -0.87 | 1.71E-04 | Unaltered group |
| EVI2A | -0.76 | 1.99E-04 | Unaltered group |
| RND2 | 0.82 | 2.04E-04 | Altered group |
| CYBB | -0.83 | 2.08E-04 | Unaltered group |
| NKAIN1 | 0.95 | 2.08E-04 | Altered group |
| DHRS9 | -1.04 | 2.13E-04 | Unaltered group |
| SLCO2B1 | -0.73 | 2.21E-04 | Unaltered group |
| TAGAP | -0.72 | 2.21E-04 | Unaltered group |
| DPYSL5 | 1.64 | 2.25E-04 | Altered group |
| FCGR3A | -0.76 | 2.29E-04 | Unaltered group |
| SIX3 | 1.78 | 2.63E-04 | Altered group |
| TUBB2A | 0.76 | 2.84E-04 | Altered group |
| LILRA2 | -0.74 | 2.97E-04 | Unaltered group |
| MT1L | -0.85 | 3.04E-04 | Unaltered group |
| TFEC | -0.74 | 3.08E-04 | Unaltered group |
| LAMA1 | 0.97 | 3.15E-04 | Altered group |
| TMEM52B | -0.77 | 3.40E-04 | Unaltered group |
| TMSB15A | 1.08 | 3.73E-04 | Altered group |
| C3AR1 | -0.71 | 3.96E-04 | Unaltered group |
| FZD2 | 0.72 | 4.06E-04 | Altered group |
| TCF7L1 | 0.8 | 4.08E-04 | Altered group |
| OLFM2 | 0.89 | 4.08E-04 | Altered group |
| HPGDS | -0.73 | 4.52E-04 | Unaltered group |
| PCED1B-AS1 | -0.73 | 5.02E-04 | Unaltered group |
| CNIH2 | 0.71 | 5.05E-04 | Altered group |
| BTK | -0.71 | 5.25E-04 | Unaltered group |
| RIPPLY3 | 0.88 | 6.33E-04 | Altered group |
| CSF1R | -0.72 | 6.57E-04 | Unaltered group |
| RTBDN | 1.04 | 6.71E-04 | Altered group |
| CLDN16 | -1.57 | 6.78E-04 | Unaltered group |
| CELF3 | 0.92 | 6.86E-04 | Altered group |
| CD300LF | -0.72 | 7.26E-04 | Unaltered group |
| FGL2 | -0.71 | 7.80E-04 | Unaltered group |
| SDK2 | 1.06 | 7.92E-04 | Altered group |
| PCYT1B | 0.84 | 8.03E-04 | Altered group |
| CTSE | -1.14 | 8.38E-04 | Unaltered group |
| CD84 | -0.76 | 8.43E-04 | Unaltered group |
| AQP9 | -0.99 | 9.13E-04 | Unaltered group |
| ELAVL3 | 0.96 | 9.18E-04 | Altered group |
| PTPN22 | -0.72 | 9.18E-04 | Unaltered group |
| MT1H | -1.34 | 9.39E-04 | Unaltered group |
| CCR2 | -0.87 | 9.77E-04 | Unaltered group |
| GFRA3 | 0.98 | 9.99E-04 | Altered group |
| MMP28 | -0.75 | 1.00E-03 | Unaltered group |
| TMSB15B | 0.78 | 1.01E-03 | Altered group |
| MT1E | -0.99 | 1.01E-03 | Unaltered group |
| ALPL | 0.8 | 1.02E-03 | Altered group |
| INA | 1.1 | 1.03E-03 | Altered group |
| PPY2P | 0.74 | 1.04E-03 | Altered group |
| LYZ | -0.81 | 1.05E-03 | Unaltered group |
| GPC2 | 0.76 | 1.05E-03 | Altered group |
| SCUBE3 | 1.09 | 1.06E-03 | Altered group |
| ARHGAP15 | -0.74 | 1.06E-03 | Unaltered group |
| DDIT4L | -0.87 | 1.06E-03 | Unaltered group |
| ELOVL4 | 0.77 | 1.08E-03 | Altered group |
| FCGR2C | -0.75 | 1.08E-03 | Unaltered group |
| HES4 | 0.73 | 1.10E-03 | Altered group |
| GLT1D1 | 0.94 | 1.12E-03 | Altered group |
| GPT | -0.8 | 1.13E-03 | Unaltered group |
| TGM1 | -0.98 | 1.16E-03 | Unaltered group |
| PTPRC | -0.73 | 1.18E-03 | Unaltered group |
| CCDC151 | 0.78 | 1.25E-03 | Altered group |
| HAPLN4 | 0.88 | 1.29E-03 | Altered group |
| TUBB2B | 1.58 | 1.31E-03 | Altered group |
| CCR5 | -0.72 | 1.32E-03 | Unaltered group |
| NCF1B | -0.75 | 1.33E-03 | Unaltered group |
| LY6G6C | -1.15 | 1.36E-03 | Unaltered group |
| BTBD17 | 1.01 | 1.42E-03 | Altered group |
| CCR4 | -0.71 | 1.42E-03 | Unaltered group |
| KCNN1 | 0.99 | 1.43E-03 | Altered group |
| VNN1 | -0.93 | 1.46E-03 | Unaltered group |
| DOCK2 | -0.75 | 1.48E-03 | Unaltered group |
| SIGLEC8 | -0.87 | 1.49E-03 | Unaltered group |
| CPT1C | 0.77 | 1.52E-03 | Altered group |
| CEL | 0.85 | 1.53E-03 | Altered group |
| SMTNL2 | 1.01 | 1.60E-03 | Altered group |
| GALR2 | 0.8 | 1.67E-03 | Altered group |
| NPTX2 | 0.92 | 1.71E-03 | Altered group |
| CECR7 | 0.98 | 1.77E-03 | Altered group |
| KCNG1 | 0.97 | 1.83E-03 | Altered group |
| FCGR2B | -0.72 | 1.85E-03 | Unaltered group |
| SLC29A4 | 0.71 | 1.91E-03 | Altered group |
| ST8SIA3 | 1.03 | 1.96E-03 | Altered group |
| SGSM1 | 0.79 | 1.98E-03 | Altered group |
| APOC2 | -0.78 | 2.07E-03 | Unaltered group |
| ITGAM | -0.72 | 2.22E-03 | Unaltered group |
| LGALS17A | -0.96 | 2.37E-03 | Unaltered group |
| MT1A | -0.91 | 2.48E-03 | Unaltered group |
| GPR82 | -0.71 | 2.58E-03 | Unaltered group |
| OLR1 | -0.8 | 2.66E-03 | Unaltered group |
| MT1M | -0.93 | 2.94E-03 | Unaltered group |
| SCT | -0.76 | 2.99E-03 | Unaltered group |
| FLRT1 | 0.74 | 3.04E-03 | Altered group |
| EMILIN3 | 0.72 | 3.12E-03 | Altered group |
| IGSF21 | -0.72 | 3.33E-03 | Unaltered group |
| MSI1 | 0.84 | 3.36E-03 | Altered group |
| DISP3 | 1.01 | 3.39E-03 | Altered group |
| CELF5 | -0.83 | 3.46E-03 | Unaltered group |
| NAPSB | -0.77 | 3.50E-03 | Unaltered group |
| OR1N1 | 0.98 | 3.54E-03 | Altered group |
| LHX2 | 0.9 | 3.58E-03 | Altered group |
| GALP | 0.76 | 3.58E-03 | Altered group |
| PHF21B | 0.86 | 3.69E-03 | Altered group |
| THBS4 | 0.9 | 3.79E-03 | Altered group |
| SNCB | 0.96 | 3.89E-03 | Altered group |
| STUM | 0.75 | 3.92E-03 | Altered group |
| PLA2G3 | 0.82 | 3.96E-03 | Altered group |
| SH3GL3 | 0.75 | 4.07E-03 | Altered group |
| ADGRG5 | -0.77 | 4.27E-03 | Unaltered group |
| KCNG3 | 0.71 | 4.43E-03 | Altered group |
| PCSK1N | 0.94 | 4.58E-03 | Altered group |
| TMEM178A | 0.73 | 4.61E-03 | Altered group |
| TBX1 | 0.91 | 4.73E-03 | Altered group |
| SLC10A4 | 0.77 | 4.74E-03 | Altered group |
| GLI1 | 0.73 | 4.83E-03 | Altered group |
| TBX4 | 0.72 | 4.88E-03 | Altered group |
| KCNF1 | 0.86 | 4.90E-03 | Altered group |
| KIF1A | 1.13 | 5.01E-03 | Altered group |
| FST | -0.89 | 5.36E-03 | Unaltered group |
| PDZK1IP1 | -0.91 | 5.40E-03 | Unaltered group |
| IL21R | -0.88 | 5.43E-03 | Unaltered group |
| SLPI | -0.75 | 5.65E-03 | Unaltered group |
| PLD4 | -0.76 | 5.74E-03 | Unaltered group |
| IGDCC3 | 1.14 | 5.79E-03 | Altered group |
| GSC | 0.87 | 5.88E-03 | Altered group |
| PCDH15 | 0.94 | 5.90E-03 | Altered group |
| CPNE7 | -0.71 | 5.93E-03 | Unaltered group |
| ALOX12B | 0.76 | 6.03E-03 | Altered group |
| SLC15A1 | 0.77 | 6.15E-03 | Altered group |
| VAX2 | 0.71 | 6.19E-03 | Altered group |
| CRISP3 | -1.28 | 6.33E-03 | Unaltered group |
| HPCAL4 | 0.74 | 6.49E-03 | Altered group |
| GABRQ | 0.76 | 6.53E-03 | Altered group |
| SNCG | -0.87 | 6.79E-03 | Unaltered group |
| ZBED2 | -0.86 | 7.12E-03 | Unaltered group |
| SFTPB | -0.83 | 7.28E-03 | Unaltered group |
| LRG1 | -0.84 | 7.33E-03 | Unaltered group |
| ST8SIA2 | 0.84 | 7.59E-03 | Altered group |
| TMEM59L | 0.89 | 7.84E-03 | Altered group |
| COL8A1 | -0.84 | 7.95E-03 | Unaltered group |
| TRIM61 | -0.71 | 8.08E-03 | Unaltered group |
| DPF1 | 0.79 | 8.76E-03 | Altered group |
| FCGBP | -0.72 | 8.77E-03 | Unaltered group |
| SIX2 | 0.88 | 8.80E-03 | Altered group |
| COL10A1 | -1.03 | 8.86E-03 | Unaltered group |
| RSPO1 | -1.21 | 9.02E-03 | Unaltered group |
| SLAMF7 | -0.73 | 9.43E-03 | Unaltered group |
| SLC34A2 | -0.79 | 9.43E-03 | Unaltered group |
| CRLF1 | 1.01 | 9.51E-03 | Altered group |
| BHMT2 | 0.75 | 9.76E-03 | Altered group |
| PAX2 | 1.65 | 1.00E-02 | Altered group |
| EPYC | -1.33 | 0.0103 | Unaltered group |
| CYP1A2 | -0.88 | 0.0106 | Unaltered group |
| MAPK4 | 0.8 | 0.0106 | Altered group |
| ATP1B2 | 0.75 | 0.0107 | Altered group |
| GPC3 | 0.82 | 0.0116 | Altered group |
| ADARB2 | 0.75 | 0.0118 | Altered group |
| VIL1 | 0.75 | 0.0118 | Altered group |
| RRAD | -0.74 | 0.0118 | Unaltered group |
| SLC17A7 | 0.77 | 0.012 | Altered group |
| ZNF683 | -0.75 | 0.0121 | Unaltered group |
| CXCL9 | -0.87 | 0.0123 | Unaltered group |
| CXCL11 | -0.76 | 0.0124 | Unaltered group |
| SLC28A3 | -0.75 | 0.0125 | Unaltered group |
| SOX11 | 1.26 | 0.0132 | Altered group |
| TMPRSS2 | 0.8 | 0.0133 | Altered group |
| FREM2 | 0.88 | 0.0134 | Altered group |
| COL25A1 | 0.8 | 0.014 | Altered group |
| PYY | 0.89 | 0.0141 | Altered group |
| KCNA6 | 0.71 | 0.0142 | Altered group |
| KIRREL2 | 0.9 | 0.0146 | Altered group |
| CD1E | -0.78 | 0.0148 | Unaltered group |
| PPP2R2C | 0.98 | 0.0149 | Altered group |
| SOX3 | 1.01 | 0.0153 | Altered group |
| COCH | 0.78 | 0.0153 | Altered group |
| SELENOV | 0.73 | 0.0159 | Altered group |
| RASSF10 | -0.72 | 0.0161 | Unaltered group |
| FSD1 | 0.83 | 0.0167 | Altered group |
| ADAMTS19 | 0.76 | 0.017 | Altered group |
| ECEL1 | 0.88 | 0.0171 | Altered group |
| ADAMDEC1 | -0.88 | 0.0171 | Unaltered group |
| ADIRF | -0.76 | 0.0175 | Unaltered group |
| GRIK4 | 0.71 | 0.0177 | Altered group |
| UPK2 | 0.73 | 0.0182 | Altered group |
| DAPL1 | -0.92 | 0.0186 | Unaltered group |
| IGF2BP1 | 0.91 | 0.0194 | Altered group |
| DPPA4 | 0.74 | 0.0197 | Altered group |
| GABRA3 | 1.04 | 0.0208 | Altered group |
| LMX1B | 0.99 | 0.0223 | Altered group |
| CSMD1 | 0.72 | 0.0226 | Altered group |
| DDX25 | 0.83 | 0.0229 | Altered group |
| IP6K3 | -0.72 | 0.0239 | Unaltered group |
| CYP2W1 | 0.79 | 0.0239 | Altered group |
| MLC1 | 0.94 | 0.0239 | Altered group |
| LCN2 | -0.72 | 0.0244 | Unaltered group |
| BRSK2 | 0.72 | 0.025 | Altered group |
| SLC7A3 | 0.78 | 0.0265 | Altered group |
| DACT2 | 0.79 | 0.0267 | Altered group |
| OPRK1 | 0.73 | 0.0277 | Altered group |
| OMD | -0.77 | 0.0283 | Unaltered group |
| CYP4B1 | -0.84 | 0.029 | Unaltered group |
| POF1B | -0.84 | 0.0304 | Unaltered group |
| FGF17 | 0.79 | 0.0344 | Altered group |
| GRIK3 | -0.97 | 0.0348 | Unaltered group |
| SFRP4 | -0.73 | 0.0348 | Unaltered group |
| CHIT1 | -0.72 | 0.0355 | Unaltered group |
| PCDHA10 | 0.86 | 0.0355 | Altered group |
| PIANP | 0.72 | 0.0367 | Altered group |
| SERPINB7 | -0.73 | 0.0397 | Unaltered group |
| ITGBL1 | -0.76 | 0.0399 | Unaltered group |
| CHGA | 0.8 | 0.041 | Altered group |
| SAMD11 | 0.79 | 0.0413 | Altered group |
| TMED7-TICAM2 | -0.82 | 0.0414 | Unaltered group |
| AGR2 | 0.78 | 0.042 | Altered group |
| SPRR1B | 0.72 | 0.0438 | Altered group |
| IGLON5 | 0.82 | 0.0445 | Altered group |
| SLC5A1 | -0.81 | 0.0448 | Unaltered group |
| NXF2 | 1.04 | 0.045 | Altered group |
| PNOC | -0.78 | 0.045 | Unaltered group |
| PI3 | -0.82 | 0.0471 | Unaltered group |
| EDN3 | 0.93 | 0.0476 | Altered group |
| PGA3 | -0.71 | 0.0483 | Unaltered group |
| NR5A1 | 0.95 | 0.0496 | Altered group |
