# Supplementary material for: Prognostic Value and Therapeutic Potential of CBX Family Members in Ovarian Cancer
Source: Front Cell Dev Biol. 2022 Jan 27;10:832354. doi: 10.3389/fcell.2022.832354 (PMC8829121; doi:10.3389/fcell.2022.832354)
Supplement: Supplementary file 6 [file Table3.DOCX]

**Supplementary Table S3.** The mRNA expression profiles of the CBXs family members in OV using the ONCOMINE database.

| **Name** | **Dataset** | **Fold change** | ***p* value** | **References** |
| --- | --- | --- | --- | --- |
| CBX1 | Bonome Ovarian | -2.560 | 3.91E-12 | [33] |
| CBX3 | TCGA Ovarian | 2.064 | 1.40E-7 | none |
| CBX7 | Bonome Ovarian | -2.937 | 5.43E-12 | [33] |
|  | TCGA Ovarian | -3.739 | 4.50E-6 | none |
|  | Yoshihara Ovarian | -5.422 | 7.48E-10 | [34] |
